# Ligand uptake in *Mycobacterium tuberculosis* truncated hemoglobins is controlled by both internal tunnels and active site water molecules

**DOI:** 10.12688/f1000research.5921.2

**Published:** 2015-07-22

**Authors:** Ignacio Boron, Juan Pablo Bustamante, Kelly S Davidge, Sandip Singh, Lesley AH Bowman, Mariana Tinajero-Trejo, Sebastián Carballal, Rafael Radi, Robert K Poole, Kanak Dikshit, Dario A Estrin, Marcelo A Marti, Leonardo Boechi

**Affiliations:** 1 Departamento de Química Biológica, Facultad de Ciencias Exactas y Naturales, Universidad de Buenos Aires, Buenos Aires, C1428EGA, Argentina; 2 Departamento de Química Inorgánica, Analítica y Química Física, INQUIMAE-CONICET, Facultad de Ciencias Exactas y Naturales, Universidad de Buenos Aires, Buenos Aires, C1428EGA, Argentina; 3 Centre for Biomolecular Sciences, The University of Nottingham, Nottingham, NG7 2RD, UK; 4 Institute of Microbial Technology, CSIR, Chandigarh, 160036, India; 5 Sir William Dunn School of Pathology, University of Oxford, Oxford, OX1 3RE, UK; 6 Molecular Biology and Biotechnology, The University of Sheffield, Sheffield, S10 2TN, UK; 7 Departamento de Bioquímica and Center for Free Radical and Biomedical Research, Facultad de Medicina, Universidad de la República, Montevideo, 11100, Uruguay; 8 Instituto de Cálculo, Facultad de Ciencias Exactas y Naturales, Universidad de Buenos Aires, Buenos Aires, C1428EGA, Argentina

**Keywords:** Mycobacterium tuberculosis, hemoglobin, water molecules, ligand interaction

## Abstract

*Mycobacterium tuberculosis,* the causative agent of human tuberculosis, has two proteins belonging to the truncated hemoglobin (trHb) family. Mt-trHbN presents well-defined internal hydrophobic tunnels that allow O _2_ and ^•^NO to migrate easily from the solvent to the active site, whereas Mt-trHbO possesses tunnels interrupted by a few bulky residues, particularly a tryptophan at position G8. Differential ligand migration rates allow Mt-trHbN to detoxify ^•^NO, a crucial step for pathogen survival once under attack by the immune system, much more efficiently than Mt-trHbO. In order to investigate the differences between these proteins, we performed experimental kinetic measurements, ^•^NO decomposition, as well as molecular dynamics simulations of the wild type *Mt-trHbN* and two mutants, VG8F and VG8W. These mutations affect both the tunnels accessibility as well as the affinity of distal site water molecules, thus modifying the ligand access to the iron. We found that a single mutation allows Mt-trHbN to acquire ligand migration rates comparable to those observed for Mt-trHbO, confirming that ligand migration is regulated by the internal tunnel architecture as well as by water molecules stabilized in the active site.

## Introduction

*Mycobacterium tuberculosis*, the causative agent of human tuberculosis, affects approximately two billion people world-wide, causing over three millions deaths each year ^1^. The genome of this pathogenic organism includes two genes, *glbN* and *glbO*, which encode for two proteins, termed here truncated hemoglobin N (Mt-trHbN) and truncated hemoglobin O (Mt-trHbO), belonging to the truncated hemoglobin (trHb) family of heme proteins, widely distributed in eubacteria, cyanobacteria, microbial eukaryotes and plants ^2, 3^.

The truncated hemoglobin family exhibits a three-dimensional structure similar to the common globin fold of myoglobin, but significantly smaller. The secondary structure of trHbs consists of four α-helices arranged in a two-over-two antiparallel sandwich instead of the common three-over-three helix globin fold. Phylogenetic analysis has distinguished three different groups of truncated hemoglobins, classified as groups I, II and III, also called N, O and P, respectively.

It has been shown that group I Mt-trHbN catalyzes the detoxification of ^•^NO in the presence of O _2_^4, 5^. The first step of this mechanism involves O _2_ migration and binding. Subsequently, ^•^NO migrates to the active site and reacts with the heme-bound O _2_ to yield an unstable peroxynitrite adduct, which isomerizes to generate the relatively innocuous nitrate anion.

**Figure 1.**
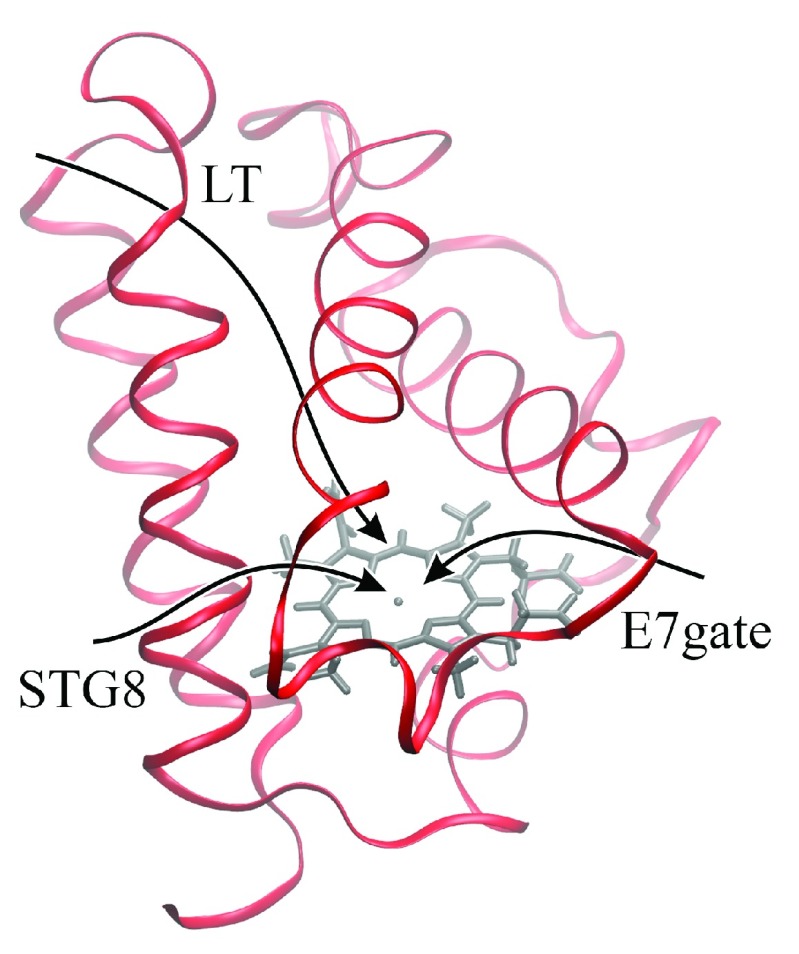
Schematic representation of the two pathways for ligand migration presented in *M. tuberculosis* trHbN. The Long Tunnel (LT) and Short Tunnel G8 (STG8) are shown in orange.

Several studies have examined the role of internal tunnels in ligand migration in trHbs ^2, 5– 8^. Three different internal tunnels have been characterize among the trHb members, in general one or two of these tunnels is found in each protein: a long tunnel (LT) topologically positioned between helices B and E, and two short tunnels, known as the E7 Gate (E7 gate) and the short tunnel G8 (STG8), which are roughly normal to the LT, as depicted in Figure 1. The E7 tunnel corresponds to the highly conserved E7 pathway widely studied in both myoglobin and hemoglobin ^9– 11^. The STG8 tunnel is analogous to that found in Mt-trHbN, next to the key residues VG8 and IH11. Previous results indicate that WG8, an absolutely conserved residue in groups II and III truncated hemoglobins, is involved in hindering ligand migration in Mt-trHbO by blocking both STG8 and LT ( Figure 1) ^12– 14^. In addition, the presence of a smaller residue at the G8 position in the Mt-trHbO mutant (WG8F) was observed to increase the small ligand association constant, although the molecular details of this process were not investigated ^12– 14^. It has also been noted that in myoglobin, *M. Tuberculosis* trHbN as well as in *T. fusca* trHbO, internal water molecules were observed to block the heme accessibility, thus delaying ligand binding ^15– 17^.

By performing CO association kinetic constant measurements ( *k _on_ CO*), ^•^NO decomposition, and molecular dynamics (MD) simulations of Mt-trHbN, we addressed molecular mechanisms that control ligand association in *M. tuberculosis* truncated hemoglobins.

## Materials and methods

### Site-directed mutant construction

The trHbN G8 mutants (VG8W and VG8F) were prepared using the Stratagene QuikChange mutagenesis kit. The following primers were designed using Primer3 http://biotools.umassmed.edu/bioapps/primer3_www.cgi ^18^ to generate single amino acid substitutions (underlined): i) WG8: forward primer 5’–CACTTCAGCCTG TGGGCCGGACACTTGG–3’ and reverse primer 5’–CAAGTGTCCGGC CCACAGGCTGAAGTG–3’; ii) FG8: forward primer 5’–ACCACTTCAGCCTG TTCGCCGGACACTTG–3’ and reverse primer 5’–CAAGTGTCCGGC GAACAGGCTGAAGTGGT–3’. Polymerase chain reaction (PCR) amplification of pET9b carrying the glbN gene with the aforementioned primers was conducted following manufacturer’s instructions. The PCR mix consisted of 5 µl 10x reaction buffer, 5–50 ng double stranded DNA template, 125 ng oligonucleotide primer 1, 125 ng oligonucleotide primer 2, 1 µl dNTP mix, 1 µl *PfuUltra* HF DNA polymerase and double distilled H _2_O to a final volume of 50 µl. The PCR reaction was 95°C for 30 s, followed by 16 cycles of: 95°C for 30 s, 55°C for 1 min and 68°C for 4 min. The reaction mix was then digested with DpnI to remove parental methylated DNA. Plasmid containing the mutated gene was then purified and used to transform *Escherichia coli* XL-1 Blue electrocompetent cells. Cells were provided by Invitrogen. Constructs were checked by sequencing.

### Protein purification

All chemicals and reagents were obtained from Sigma Aldrich, unless indicated otherwise. The trHbN protein variants were purified using standard techniques reported for other bacterial globins ^19^. Briefly, mutated constructs were used to transform *E. coli* BL21 DE3 pLysS. Starter cultures grown overnight in LB supplemented with kanamycin (50 μg ml ^-1^) and chloramphenicol (35 μg ml ^-1^) were used to inoculate 6 batches of 1 L LB medium at 1% (v/v), supplemented with kanamycin and 3 μM FeCl _3_. Once cultures reached an OD _600_ of around 0.4, expression of trHbN was initiated by the addition of 1 mM IPTG and grown for a further 4 h. Cells were harvested by centrifugation at 5500 rpm for 20 min at 4°C and stored overnight at -20°C. After thawing, cells were resuspended in 40 ml buffer (10 mM TRIS-HCl (pH 7.0) with 1 mM EDTA, 10 mM DTT, 45 μg ml ^-1^ phenylmethylsulphonyl fluoride, 500 μg ml ^-1^ RNase and 50 μl DNase), homogenized using a Douce homogeniser and ultracentrifuged at 44,000 rpm for 1 h at 4°C. The supernatant, red in color, was loaded onto a 30 ml DEAE Sepharose Fast Flow column (Pharmacia Biotech) equilibrated with 10 mM TRIS-HCl (pH 7.0), washed with the same buffer until the UV trace returned to baseline, and eluted via a gradient from 0 to 1 M NaCl in 10 mM TRIS-HCl using an Akta purifier (GE Healthcare Bio-Sciences, Amersham Biosciences, U.K. Ltd.). Fractions that were most red in color were concentrated using a Vivaspin 20 concentrator (Sartorius Stedim Biotech) to around 5 ml and loaded onto a gel filtration Superdex 75 column, equilibrated with 0.15 M NaCl in 10 mM TRIS-HCl (pH 7.5); again, fractions with the most color were collected, combined and stored at -80°C. Purity was checked using gel electrophoresis and analysis of the heme-to-protein ratio (410 nm and 280 nm in the UV-visible absorption spectrum).

### Kinetic stopped flow measurements of CO binding

Rapid mixing experiments were conducted with a thermostated stopped flow apparatus (BioLogic SFM-300). Kinetics of carbon monoxide (CO) binding to determine the *k _on_ CO* were measured on the deoxy state of mutant and wild type globins at 20ºC. Solutions containing 5 μM protein in a 100 mM sodium phosphate at pH 7.0 were degassed in a nitrogen atmosphere and reduced with an equimolar concentration of sodium dithionite and mixed with increasing CO concentrations. The observed pseudo first-order rate constant ( *k _obs_*) was determined by fitting the absorbance decay resulting from association of the protein with CO, to a single exponential function. Kinetic rate constants ( *k _on_ CO*) were obtained from the slope of the plots of *k _obs_* as a function of CO concentration.

### ^•^NO decomposition

To determine rates of nitric oxide ( ^•^NO) decomposition by wild type and mutant Mt-trHbN proteins, ^•^NO was added, as ProliNONOate, to a solution of 50 mM KPi buffer with 50 μM EDTA (pH 7.5), 100 μM NADPH and 100 nM *E. coli* ferredoxin reductase inside a thermoregulated, magnetically stirred reaction vessel. Mt-trHbN (2 μM) was added at the apex of the signal response to 2 μM ProliNONOate and ^•^NO decay was followed until depleted using an ^•^NO electrode (World Precision Instruments). Rates of ^•^NO decay were calculated for each protein by determining the time taken for peak ^•^NO concentrations to decay by 0.5 μM and were expressed per μM heme, determined spectrally by the peak in the Soret region at 410 nm.

### Set up of the simulations

The starting structure corresponds to Mt-trHbN crystal structure (PDB entry 1IDR http://www.rcsb.org/pdb/explore/explore.do?structureId=1IDR), at 1.9 Å of resolution ^20^. The protonation state of the amino acids was assumed based on the environment of the residues in the crystal structure. All solvent-exposed His residues were protonated at the N-δ atom, as well as the proximal HisF8, because of its coordination to the heme iron. An octahedral box of 10 Å of radius, which corresponds to 5234 explicit water molecules was added to the system. TIP3P water molecules were used by tLEaP module of the AMBER12 package ^21^. The param99 Amber force field was used for all the aminoacid parameters ^22^ except heme parameters which were developed in our group ^23^ and strongly validated for being used in several studies of heme proteins ^24– 30^. Periodic boundary conditions were used for all the simulations performed with a 9 Å cutoff. Particle mesh Ewald (PME) summation method for treating the electrostatic interactions. The SHAKE algorithm was used to keep constant the non-polar hydrogen equilibrium distance. Temperature and pressure were kept constant with Langevin thermostat and barostat, respectively, as implemented in the AMBER12 program ^21^. The equilibration simulation protocol was performed as follow: (i) slowly heating the system from 0 to 300K for 20 ps at constant volume, by using harmonic restraints of 80 kcal/mol A ^2^ for all C _α_ atoms and (ii) pressure equilibration of the whole system during 1 ns at 300K with restrained atoms in (i). (iii) Unconstrained 100 ns molecular dynamics simulation at constant temperature (300K) was performed.

**Figure S1.**
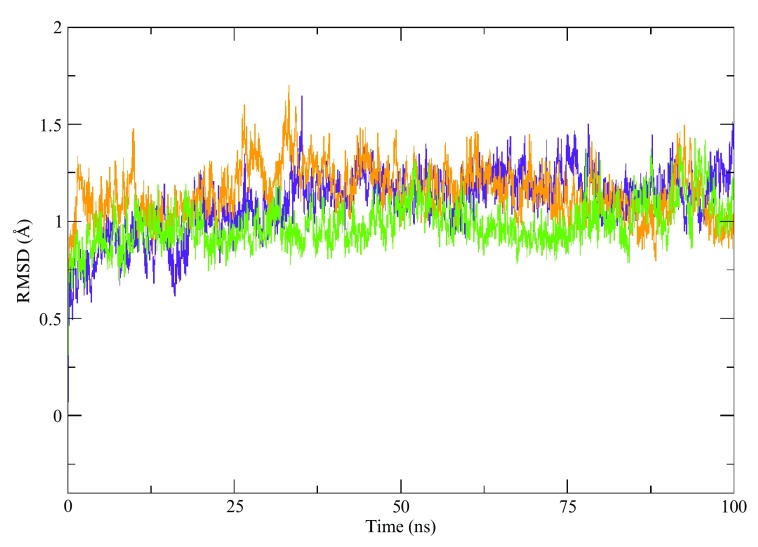
Root Mean Square Deviation during MD simulations for all the Mt-trHbN studied: wild type (green), VG8F (orange) and VG8W (violet).

*In silico* mutant proteins were built by using tLEaP module of AMBER12 package ^21^, and underwent the same protocol used for wild type protein. Root Mean Square Deviation (RMSD) was used as structure stability controls. All structures were observed to be stables during the time scale of the simulation ( Figure S1).

### Ligand migration free energy profiles

The free energy profile for the CO migration process inside the protein tunnel/cavity system was computed by the Implicit Ligand Sampling (ILS) approach that post-processes, using a probe molecule, an MD simulation performed in the absence of the ligand. This method was thoroughly tested for heme proteins ^32^. ILS calculations were performed on a rectangular grid (0.5 Å resolution) that includes the whole simulation box (i.e. protein and the solvent) and the probe used was a CO molecule. Calculations were performed on 5000 frames taken from the last 90 ns of simulation time. The values for grid size, resolution and frame numbers were tested in a previous study ^32^. Analysis of the ILS data was performed using an *ad-hoc* fortran-90 program available upon request ^32^. Moreover, ILS has been shown to yield quantitative results for ligand migration processes when compared with more costly free energy methods that treat the ligand explicitly.

## Results

### CO association kinetic constant measurements

**Figure 2.**
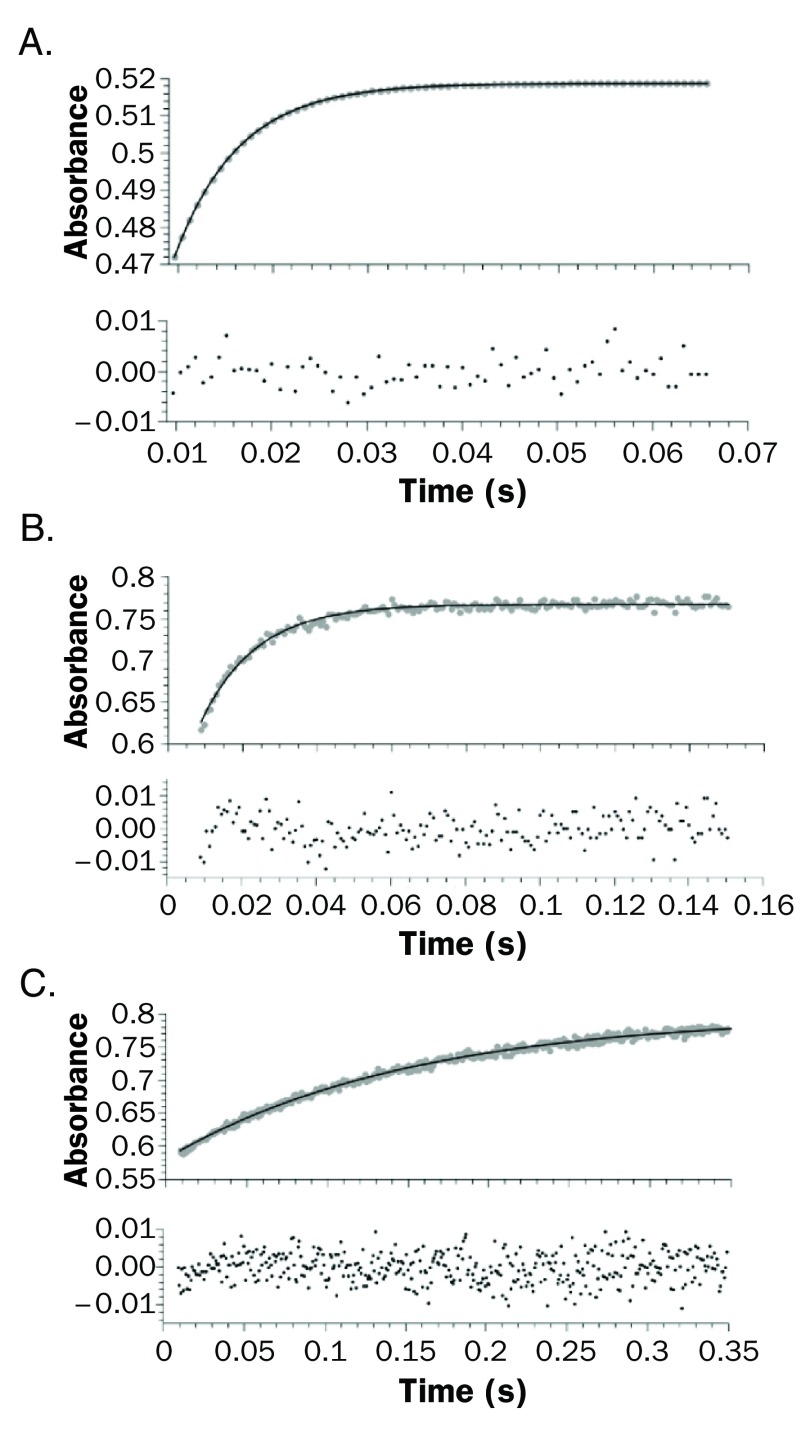
Stopped-flow time course for the reaction of reduced 5 μM wild type ( **A**), VG8F ( **B**), and VG8W ( **C**) mutants in 100 mM phosphate buffer at pH 7. The reaction was monitored at 423 nm (grey dots) and the line shows the best first order fit.

**Figure 3.**
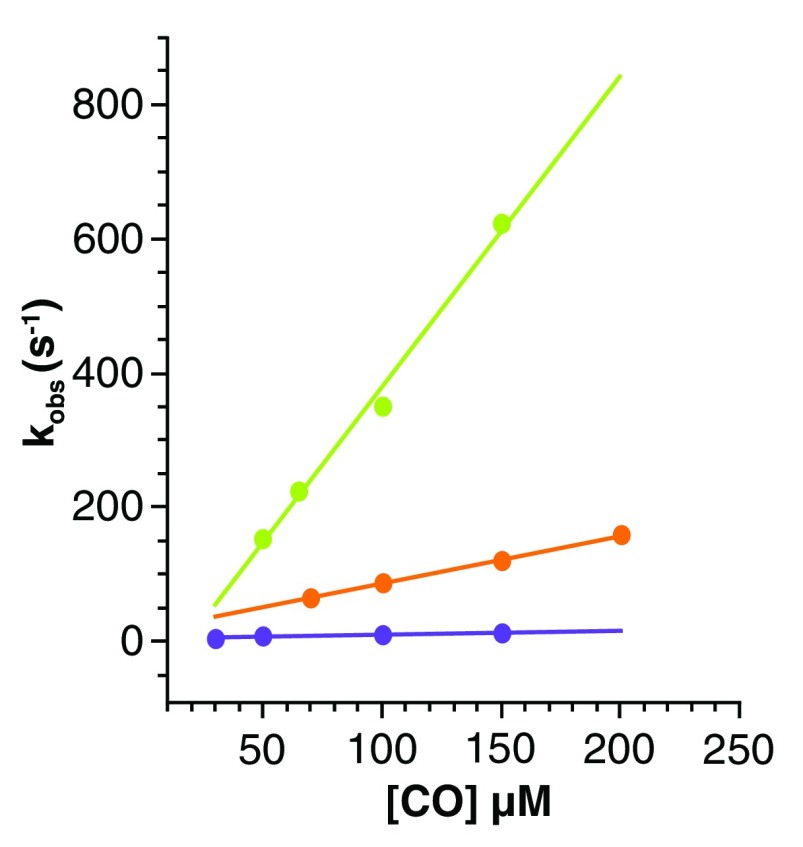
Apparent rates *k _obs_* for CO binding to ferrous Mt-trHbN. Curves for wild type (green), VG8F (orange) and VG8W (violet) mutants as a function of CO concentration in stopped flow measurements are shown. The time courses are measured at different CO concentrations ranging from 10 to 200 µM (after mixing). Continuous line corresponds to linear fit of *k _obs_* rates.

Although CO is not the natural ligand of the hemeproteins, it is widely used as a probe for ligand association studies due to its ease of use. In order to address the molecular determinants controlling ligand migration we performed CO ligand association constant measurements of wild type Mt-trHbN and two mutants: VG8F and VG8W. Kinetic traces for CO binding were measured through the absorption changes at the CO adduct peak position (λ=423 nm; Figure 2). Association of CO is well described by a single exponential decay, whose rate constant ( *k*_obs_) depends linearly on CO concentration and the slope can be interpreted as *k _on_ CO*. A significant *k _on_ CO* decrease for VG8F (715 ± 27 mM ^-1^s ^-1^), and an even larger decrease for VG8W (48 ± 1 mM ^-1^s ^-1^) was observed in relation to that observed for the wild type protein (4495 ± 357 mM ^-1^s ^-1^) ( Figure 3). Table 1 summarizes the measured *k _on_ CO* values for wild type and mutant Mt-trHbs O and N, and is presented alongside literature data.

**Table 1.** Association kinetic constants for wild type and mutants of Mt-trHbN and Mt-trHbO.

Protein	*k _on_ CO* (mM ^-1^s ^-1^)	Reference
Mt-trHbN	4495	This work
Mt-trHbN VG8F	715	This work
Mt-trHbN VG8W	48	This work
Mt-trHbO	13 (79%) - 180 (21%) *	33
Mt-trHbO WG8F	3700 (75%) - 1200 (25%) *	12

*major and minor rate contributions to a biphasic fitting are indicated between brackets.

### Molecular dynamics simulations

Small ligand association in the trHb family is presumably regulated by two main processes: i) ligand migration from solvent bulk to the protein distal site cavity, ii) displacement of water molecules from the distal site cavity ^15– 17, 35^. With this in mind, we performed classical MD simulations as they allow us to investigate both processes involved in ligand association. Ligand migration was studied using ILS calculations for the wild type, as well as both VG8F and VG8W mutant proteins. Displacement of retained water molecules in the distal site was considered by performing classical MD simulations and analyzing the solvation structure in each active site.

**Figure 4.**
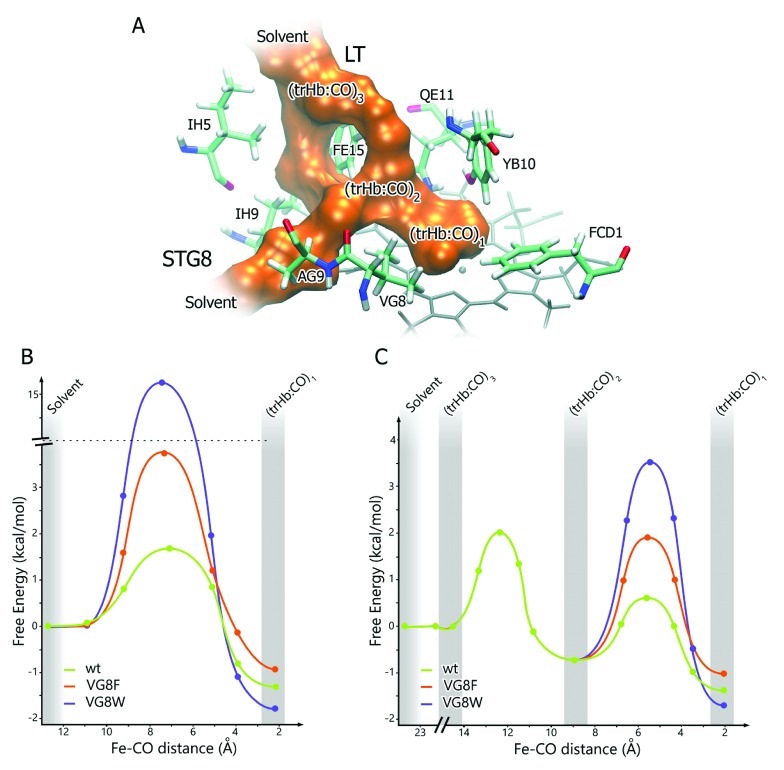
CO ligand migration along possible pathways in Mt-trHbN. ( **A**) Schematic representations of the residues involved in the heme distal site and tunnels, the two tunnels and cavities estimated with ILS for the wild type form. Free energy profiles over STG8 ( **B**) and LT ( **C**) connecting the solvent with the distal site through the cavities (trHb : CO) _1_, (trHb : CO) _2_ and (trHb : CO) _3_, for wild type (green), VG8F (orange) and VG8W (violet) mutant Mt-trHbN. Circles represent calculated free energy values with the ILS method and lines correspond to a fitting estimation of these calculated values. The x coordinate represents the Fe-CO distance along the pathways.

The wild type Mt-trHbN presents two tunnels available for ligand migration, the LT and the STG8 ( Figure 4A). On the one hand, the LT connects three internal cavities: (trHb : CO) _1_, (trHb : CO) _2_ and (trHb : CO) _3_. The STG8, on the other hand, has the distal site cavity (trHb : CO) _1_ connected to both the cavity (trHb : CO) _2_ and the solvent, although the cavity (trHb : CO) _2_ plays only a secondary role, due to the fact that it does not alter the energy migration profile along the STG8 straight from the distal cavity to the solvent. The VG8F mutant conserves both tunnels, although they are constrained compared to those in the wild type. In the VG8W case, however, the energy profiles suggest a completely blocked STG8 and a LT for which the accessibility to the iron heme is partially reduced.

In order to quantify the contribution of the single G8 mutation we computed free energy profiles for CO migration through both LT and STG8 tunnels ( Figure 4B, 4C). The free energy was set to a value of 0 kcal/mol where CO ligand is fully solvated- at 13 Å and 24 Å from the Fe atom, for STG8 and LT respectively. Wild type Mt-trHbN presents small barriers (~2 kcal mol ^-1^) for CO migration from the solvent to the active site cavity (trHb : CO) _1_ through both tunnels.

**Figure 5.**
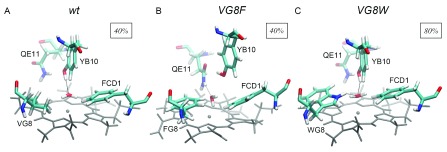
Schematic representations of the distal site of Mt-trHbN. ( **A**) wild type, ( **B**) VG8F and ( **C**) VG8W forms showing, on the basis of MD simulations, the hydrogen-bond network (dotted lines) stabilizing a water molecule above the iron heme. The percentages depicted as insets in the figure correspond to active site water occupancy during MD simulation.

The active site water molecules occupancy was computed for all three systems by performing 200 ns of MD simulations with explicit water molecules. In each case a water molecule was able to access the active site and was stabilized by the iron and the distal site residues ( Figure 5). Specifically, in both wild type and VG8F Mt-trHbN a water molecule was present for approximately 40% of the length of the simulation ( Figure 5A, 5B). The VG8W mutant active site, on the other hand, is occupied by water molecules in 80% of the simulation time, probably due to the hydrogen bonding capacity of W ( Figure 5C).

### ^•^NO decomposition in the presence of *M. tuberculosis* HbN

**Figure 6.**
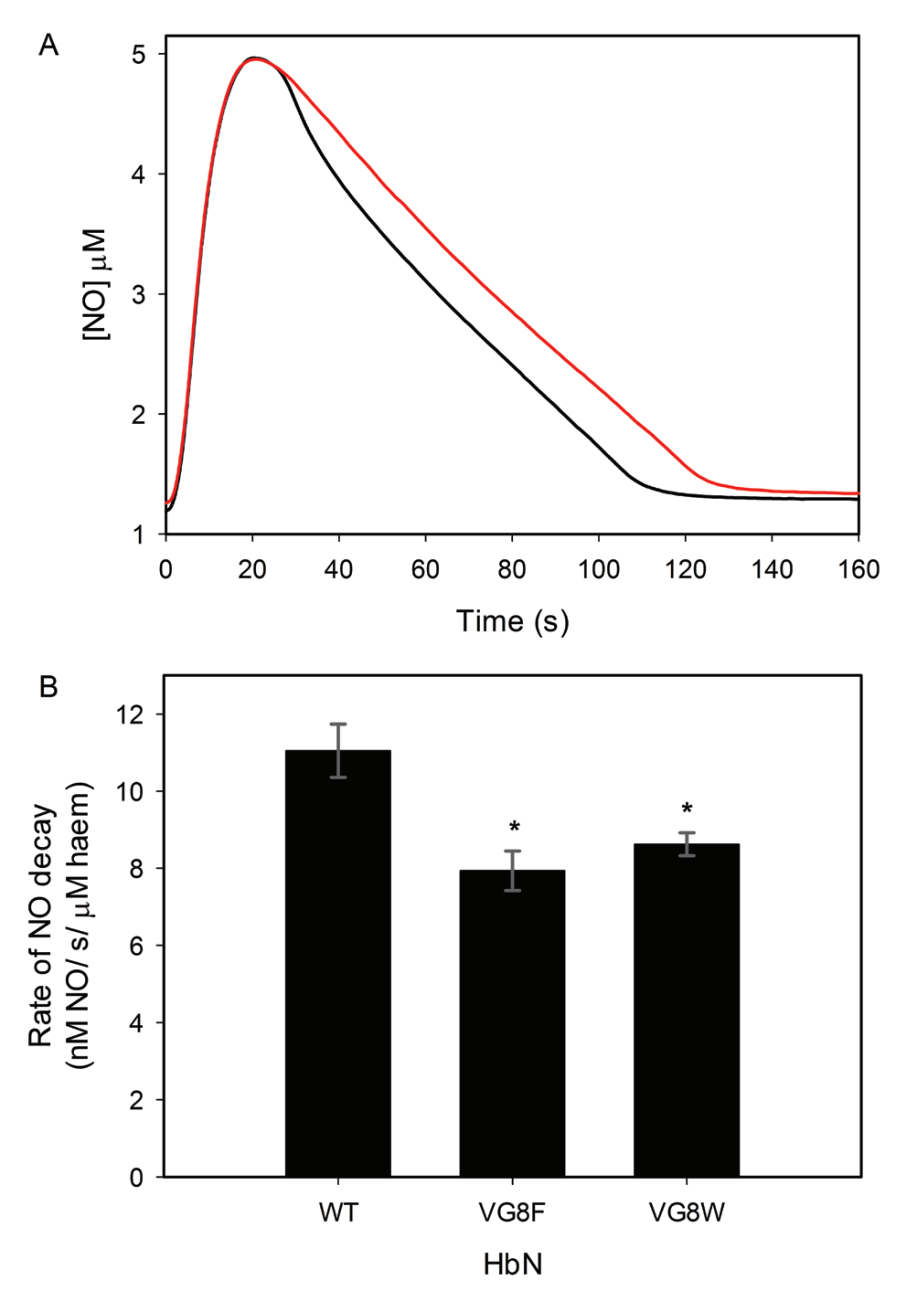
^•^NO decomposition by Mt-trHbN at ambient oxygen concentrations (approx. 200 μM, not measured). ( **A**) ^•^NO decay was monitored amperometrically in the absence (red trace) and the presence (black trace) of Mt-trHbN added at the apex of the signal response to 2 μM ProliNONOate. Data are representative of 3 technical repeats. ( **B**) Mean rates of ^•^NO decay in the presence of wild type Mt-trHbN or site-directed mutants from 3 technical repeats ± S.E.M * *P* < 0.05, unpaired *t*-test.

Mt-trHbN has previously been described as a dioxygenase, capable of O _2_-dependent ^•^NO consumption ^36, 37^. Consequently, ^•^NO decomposition by purified Mt-trHbN and the VG8F, VG8W mutants was determined in a reaction mixture containing buffer, NADPH and *E. coli* FdR, to enable cyclic restoration of heme iron to the oxyferrous state. Figure 6A shows that in the absence of protein (red trace) decay of the ^•^NO signal was monophasic until ^•^NO was exhausted. The decay of NO in the presence of Mt-trHbN (black trace) was biphasic, with an almost linear initial rapid rate in decay, which was used to compare the various Mt-trHbN derivatives, followed by a slower rate in decay. This suggests that ^•^NO is not being turned over in a cyclic manner, but is simply binding available heme. The chemical step being measured in this assay is the reaction between ^•^NO and the oxyferrous heme; once this reaction has concluded, we assume that the heme is restored from ferric back to ferrous. We are unsure why the reaction is single turnover but it could be due to (a) rapid binding of ^•^NO to the ferrous complex before oxygen can bind, rendering it unable to bind oxygen and initiate the reaction or (b) due to slow reduction of Mt-trHbN by the non-native *E. coli* FdR. ^•^NO consumption results show that the VG8F and VG8W mutants have a statistically significant reduced ^•^NO binding capacity compared to HbN ( Figure 6B).

Experimental and theoretical calculations raw data Detailed legends describing the raw data can be found in the text file provided. Click here for additional data file. Copyright: © 2015 Boron I et al. 2015 Data associated with the article are available under the terms of the Creative Commons Zero "No rights reserved" data waiver (CC0 1.0 Public domain dedication).

## Discussion

CO association kinetic constant measurements as well as MD simulations of Mt-trHbN wild type and site-specific mutants were performed to analyze the role of tunnels and water molecules in the ligand association process. ILS calculations showed that the main tunnels of wild type Mt-trHbN, STG8 and LT, were partially blocked in the VG8F mutant and STG8 was nearly completely blocked in the VG8W mutant. The analysis of water molecules showed that VG8W increases the probability of the presence of a water molecule in the distal site, which may interfere with the association process. Consistently, the association kinetic constants of CO for both Mt-trHbN mutants showed a decrease of slightly less than one order of magnitude when VG8 is replaced with F and two orders of magnitude when VG8 is replaced with W. Moreover, our data also showed that both mutants have less capacity of ^•^NO binding than wild type Mt-trHbN.

Interestingly, the Mt-trHbN VG8W mutant presents a similar *k _on_* CO to the wild type Mt-trHbO, showing that a single residue is responsible for the differential accessibility in these proteins. The results support the idea that STG8 and LT are the main channels for CO migration in the deoxygenated Mt-trHbN, as blocking these tunnels decreases the ability of CO to access the heme pocket. Although in both the mutated Mt-trHbN and wild type Mt-trHbO the STG8 is blocked by WG8, the LT remains open in Mt-trHbN, allowing CO access into the heme cavity, whereas the main tunnel for CO migration in Mt-trHbO is the E7, as was previously described ^7^. This fact shows that although the *k _on_* CO from mutant trHbN and wild type trHbO members are very similar, the ligand enters through different pathways, evidencing the complexity of mechanisms that regulate the ligand association process in these proteins.

## Data availability

The data referenced by this article are under copyright with the following copyright statement: Copyright: © 2015 Boron I et al.

Data associated with the article are available under the terms of the Creative Commons Zero "No rights reserved" data waiver (CC0 1.0 Public domain dedication). 

*F1000Research*: Dataset 1. Experimental and theoretical calculations raw data, 10.5256/f1000research.5921.d42091 ^39^
