# Supplementary material for: Ligand uptake in Mycobacterium tuberculosis truncated hemoglobins is controlled by both internal tunnels and active site water molecules
Source: F1000Res. 2015 Jul 22;4:22. Originally published 2015 Jan 23. [Version 2] doi: 10.12688/f1000research.5921.2 (PMC4591903; doi:10.12688/f1000research.5921.2)
Supplement: Peer review history

# Peer review: Referee response for version 2

**DOI:** 10.5256/f1000research.7339.r10500

**Published:** 2015-09-25

**Authors:** Michael Wilson

**Affiliations:** 1 School of Biological Sciences, University of Essex, Colchester, UK

The authors have responded positively to my earlier comments and in my view have improved the manuscript.

I have read this submission. I believe that I have an appropriate level of expertise to confirm that it is of an acceptable scientific standard.

# Peer review: Referee response for version 1

**DOI:** 10.5256/f1000research.6326.r7848

**Published:** 2015-04-24

**Authors:** Michael Wilson

**Affiliations:** 1 School of Biological Sciences, University of Essex, Colchester, UK

This is a succinct and generally well written paper reporting experimental data on ligand binding to Mt-trHbN and to two mutants (VG8F anndVG8W)).These latter have been designed to test hypotheses regarding the possible routes by which access is gained to the heme by small neutral gaseous molecules. The experiments and the molecular modelling that supports them, and which provides mechanistic insights, have been carefully performed. The results are of interest to the field as they add to the body of accumulated evidence that proteins, including those with the function of binding small neutral molecules, provide specific, and often dynamic, channels to permit rapid access to the binding site. Furthermore the kinetics of binding are seen to be strongly influenced by single amino acid substitution in the access channels.

Although the results support the general conclusions drawn by the authors some clarification of a number of points would be helpful. These are given below.

 

1. Why in Figs 2A and B does time appear not to start at t=0?
2. In Fig and discussion is it proposed that the water molecule is bound to the iron (common for ferric but not for ferrous iron) or stabilised in that location only by hydrogen binding. It is presumed that for the modelling the iron is in the ferrous state as the authors are discussing CO binding.
3. Although the authors discuss the decomposition of NO catalysed by Mt-trHbN in the presence of oxygen the assays do not make it clear that this is the reaction under study. No mention of the oxygen concentration is made in the legend to Fig 6. It seems oxygen is present to account for the disappearance of NO. From Fig 6 it is stated that NO binds to the protein but is not degraded (e.g. to nitrate via peroxynitrite). Is this because regeneration of the reduced heme (necessary for oxygen binding) is so slow or is it because as ferric heme is reduced by the NADPH/ferredoxin system NO binds before oxygen and thus no turnover occurs as it is known that the NO-ferrous complex does not react with oxygen? In any case it is not made clear to what chemical step the measured kinetics refer. It would improve the manuscript if the authors clarified these points.

I have read this submission. I believe that I have an appropriate level of expertise to confirm that it is of an acceptable scientific standard, however I have significant reservations, as outlined above.

# Response

**Published:** 2015-07-13

**Authors:** Leonardo Boechi

**Affiliations:** University of Buenos Aires, Argentina

1. *Why in Figs 2A and B does time appear not to start at t=0?*
  We thank the reviewer for noticing that specific issue. We decided to discard the first 8ms because they were too noisy.
2. *In Fig and discussion is it proposed that the water molecule is bound to the iron (common for ferric but not for ferrous iron) or stabilised in that location only by hydrogen binding. It is presumed that for the modelling the iron is in the ferrous state as the authors are discussing CO binding. *
  We agree with the reviewer that the water bounds in general very weak to the ferrous iron. However, there is evidence showing that in a polar distal site (with polar residues), as in the case of truncated hemoglobins, water molecules remain inside stabilized by the polar residues, and thus slow ligand binding to ferrous iron ( Olson and Phillips Jr, 1997; Ouellet *et al.,* 2008).
3. *Although the authors discuss the decomposition of NO catalysed by Mt-trHbN in the presence of oxygen the assays do not make it clear that this is the reaction under study. No mention of the oxygen concentration is made in the legend to Fig 6. It seems oxygen is present to account for the disappearance of NO. From Fig 6 it is stated that NO binds to the protein but is not degraded (e.g. to nitrate via peroxynitrite). Is this because regeneration of the reduced heme (necessary for oxygen binding) is so slow or is it because as ferric heme is reduced by the NADPH/ferredoxin system NO binds before oxygen and thus no turnover occurs as it is known that the NO-ferrous complex does not react with oxygen? In any case it is not made clear to what chemical step the measured kinetics refer. It would improve the manuscript if the authors clarified these points.*
  As suggested by the review we modified the caption of Figure 6, and also we add a sentence.
  The legend of the caption can be changed to:
  Figure 6. •NO decomposition by Mt-trHbN at ambient oxygen concentrations (approx. 200 µM, not measured). (A) •NO decay was monitored amperometrically in the absence (red trace) and the presence (black trace) of Mt-trHbN added at the apex of the signal response to 2 μM ProliNONOate. Data are representative of 3 technical repeats. (B) Mean rates of •NO decay in the presence of wild type Mt-trHbN or site-directed mutants from 3 technical repeats ± S.E.M *P < 0.05, unpaired t-test.
  The sentence added is:
  The chemical step being measured in this assay is the reaction between •NO and the oxyferrous heme; once this reaction has concluded, we assume that the heme is restored from ferric back to ferrous. We are unsure why the reaction is single turnover but it could be due to (a) rapid binding of •NO to the ferrous complex before oxygen can bind, rendering it unable to bind oxygen and initiate the reaction or (b) due to slow reduction of Mt-trHbN by the non-native *E. coli* FdR.

# Peer review: Referee response for version 1

**DOI:** 10.5256/f1000research.6326.r7468

**Published:** 2015-02-09

**Authors:** Marco Nardini

**Affiliations:** 1 Department of Biosciences, University of Milan, Milan, Italy

This manuscript describes CO association kinetic constant measurements, ^●^NO decomposition, and molecular dynamics simulations on the wild type truncated Hb from *Mycobacterium tuberculosis* (Mt-trHbN) and two mutants (VG8F and VG8W) which introduce modifications in the two-tunnel system of the protein. The data are then compared to those from Mt-trHbO, suggesting that ligand migration is regulated by the internal tunnel architecture as well as by water molecules stabilized in the active site.

Although the topic of the structure and the dynamic behavior of protein matrix tunnels in truncated Hb, and in particular in Mt-trHbN, has been “squeezed” a lot during the past years, the data reported in this paper add some new information and might be of interest in the field. The paper is well written (with regards to the requirements of the journal) and describes a technically sound piece of scientific research with data that supports the conclusions. Indexing is recommended, if the (few) minor comments below are addressed.

Minor remarks:

- Abstract: line 4
  The authors write that “Mt-trHbO” possesses tunnels that are partially blocked ..” In fact normally trHbOs are associated with internal discrete cavities and not with tunnels. The authors should rephrase the sentence.
- Abstract: line 11
*Mt-trHbN* should not be in Italics.
- Abstract: line 12
  The sentence “mutations introduce modifications in both tunnel topologies” is quite cryptic and it is not clear what the author mean with “tunnel topologies”. The authors should rephrase the sentence to clarify it.
- Introduction: page 3, first column, line 8
  The authors might want to include a review on trHbs more recent than that indicated in reference (3). There are several of them published in the last few years.
- Introduction: page 3, first column, line 23
  The paragraph starting from line 23 is a bit misleading because the authors try to generalize the description of the protein matrix tunnels in trHbs by mixing what happens in trHbNs and trHbOs. This is confusing since it might give the impression that three tunnels co-exist in trHbs, which is not true. In this respect, Figure 1 contributes a lot to make confusion, since it is not clear which trHb protein represents and it seems that it has three co-existing tunnels. It is probably better to keep separate trHbNs and trHbOs, both in the text description and in Figure 1. The authors should describe the tunnel features in trHbN (short and long tunnel) and trHbO (cavities, small E7 residues an possible E7 gating), and show two panels in Figure 1 with depicted the tunnel/cavity systems in Mt-trHbN (panel A) and Mt-trHbO (panel B), possibly using a similar protein orientation and highlighting the role of the G8 residue in the two cases.
- Introduction: page 3, second column, line 7
  The sentence regarding the “internal water molecules” is too generic as it is written now, since it is not clear if the authors refer to globins, to trHbs or to Mt-trHbs. The authors should rephrase the sentence to clarify this issue.
- Introduction: page 3, second column, line 10
  The authors should say that the experimental measurements and the MD simulations have been performed only on Mt-trHbN and mutants, and not, for instance, on Mt-trHbO.
- Materials and Methods: page 3, second column, line 40
  The purification paragraph seems to refers only to trHbN. What about its mutants? The authors should add a sentence to clarify this issue.
- Materials and Methods: page 4, first column, line 44
  The following sentence is not written fully correctly:
  “Amino acids protonation states were assumed based on environment of the residue in the crystal structure. All solvent-exposed His were protonated at the N- δ delta atom, as well as HisF8, because of its coordination to the heme iron”.
  One possibility is to rephrase it as follows:
  “The protonation state of the amino acids was assumed based on the environment of the residues in the crystal structure. All solvent-exposed His residues were protonated at the N-δ atom, as well as the proximal HisF8, because of its coordination to the heme iron”.
- page 5, second column, line 12
  It is not clear what the authors mean when they write that the STG8 “has only the distal site cavity, (trHb : CO) _1_, …”, especially if this sentence is coupled with Figure 4A, where (trHb : CO) _1_ seems to be connected to STG8 through (trHb : CO) _2_.
- page 5, Title of Table 1
  It is probably better to change “..for wild type and mutants Mt-trHbs O and N” to “..for wild type and mutants of Mt-trHbN and Mt-trHbO”
- page 6, Figure 4 legend
  In the legend of Panel C there is no mention of the (trHb : CO) _3_ site. 
- References: page 9
  Reference (21) is missing the title

I have read this submission. I believe that I have an appropriate level of expertise to confirm that it is of an acceptable scientific standard.

# Response

**Published:** 2015-07-13

**Authors:** Leonardo Boechi

**Affiliations:** University of Buenos Aires, Argentina

- *Abstract: line 4*
*The authors write that “Mt-trHbO” possesses tunnels that are partially blocked ..” In fact normally trHbOs are associated with internal discrete cavities and not with tunnels. The authors should rephrase the sentence.*
  We rephrase the sentence “whereas Mt-trHbO possesses tunnels that are partially blocked by a few bulky residues, particularly atryptophan at position G8” by writing “whereas Mt-trHbO possesses tunnels interrupted by a few bulky residues, particularly a tryptophan at position G8”
- *Abstract: line 11*
*Mt-trHbN should not be in Italics.*
  Corrected, thanks 
- *Abstract: line 12*
*The sentence “mutations introduce modifications in both tunnel topologies” is quite cryptic and it is not clear what the author mean with “tunnel topologies”. The authors should rephrase the sentence to clarify it.*
  We rephrase the sentence “These mutations introduce modifications in both tunnel topologies and affect the incoming ligand capacity to displace retained water molecules at the active site.” By writing “These mutations affect both the tunnels accessibility as well as the affinity of distal site water molecules, thus modifying the ligand access to the iron”
- *Introduction: page 3, first column, line 8*
*The authors might want to include a review on trHbs more recent than that indicated in reference (3). There are several of them published in the last few years.*
  As suggested by the review we modified the references by more recent ones: Davidge & Dikshit (2013).
- *Introduction: page 3, first column, line 23*
*The paragraph starting from line 23 is a bit misleading because the authors try to generalize the description of the protein matrix tunnels in trHbs by mixing what happens in trHbNs and trHbOs. This is confusing since it might give the impression that three tunnels co-exist in trHbs, which is not true. In this respect, Figure 1 contributes a lot to make confusion, since it is not clear which trHb protein represents and it seems that it has three co-existing tunnels. It is probably better to keep separate trHbNs and trHbOs, both in the text description and in Figure 1. The authors should describe the tunnel features in trHbN (short and long tunnel) and trHbO (cavities, small E7 residues an possible E7 gating), and show two panels in Figure 1 with depicted the tunnel/cavity systems in Mt-trHbN (panel A) and Mt-trHbO (panel B), possibly using a similar protein orientation and highlighting the role of the G8 residue in the two cases.*
  As suggested by the review, we modified the sentence to clarify.
  “Three internal tunnels were found in the truncated hemoglobin family:” by “Three different internal tunnels have been characterize among the trHb members, in general one or two of these tunnels is found in each protein:”
  We also change Figure 1 and its caption as suggested.
  Caption Figure 1. Schematic representation of the two pathways for ligand migration presented in *M. tuberculosis* trHbN. The Long Tunnel (LT) and Short Tunnel G8 (STG8) are shown in orange.
- *Introduction: page 3, second column, line 7*
*The sentence regarding the “internal water molecules” is too generic as it is written now, since it is not clear if the authors refer to globins, to trHbs or to Mt-trHbs. The authors should rephrase the sentence to clarify this issue.*
  As suggested by the review we clarify the sentence: “It has also been noted that in myoglobin, M. Tuberculosis trHbN as well as in T. fusca trHbO, internal water molecules were observed to block the heme accessibility, thus delaying ligand binding”
- *Introduction: page 3, second column, line 10*
*The authors should say that the experimental measurements and the MD simulations have been performed only on Mt-trHbN and mutants, and not, for instance, on Mt-trHbO.*
  As suggested by the review we clarify the sentence by adding explicitly the name of the protein studied “By performing CO association kinetic constant measurements (…) of Mt-trHbN, we addressed molecular mechanisms that control ligand association in M. Tuberculosis truncated hemoglobins” 
- *Materials and Methods: page 3, second column, line 40*
*The purification paragraph seems to refers only to trHbN. What about its mutants? The authors should add a sentence to clarify this issue.*
  We clarify this by modifying “The trHbN protein was” by “The trHbN protein variants were”  
- *Materials and Methods: page 4, first column, line 44*
*The following sentence is not written fully correctly:*
*“Amino acids protonation states were assumed based on environment of the residue in the crystal structure. All solvent-exposed His were protonated at the N- δ delta atom, as well as HisF8, because of its coordination to the heme iron”.*
*One possibility is to rephrase it as follows:*
*“The protonation state of the amino acids was assumed based on the environment of the residues in the crystal structure. All solvent-exposed His residues were protonated at the N-δ atom, as well as the proximal HisF8, because of its coordination to the heme iron”.*
  We thank the reviewer for the suggestion, the phrase was modified as suggested.
- *page 5, second column, line 12*
*It is not clear what the authors mean when they write that the STG8 “has only the distal site cavity, (trHb : CO) _1_, …”, especially if this sentence is coupled with Figure 4A, where (trHb : CO) _1_ seems to be connected to STG8 through (trHb : CO) _2_.*
  We modified the sentence by adding information:
  On the one hand, the LT connects three internal cavities: (trHb : CO) 1 , (trHb : CO) 2 and (trHb : CO) 3 . The STG8, on the other hand, connected to both the cavity (trHb : CO) 2 and the solvent.
- *page 5, Title of Table 1*
*It is probably better to change “..for wild type and mutants Mt-trHbs O and N” to “..for wild type and mutants of Mt-trHbN and Mt-trHbO”*
  We thank the reviewer for the suggestion; the phrase was modified as suggested.
- *page 6, Figure 4 legend*
*In the legend of Panel C there is no mention of the (trHb : CO) _3_ site.*
  We thank the reviewer for the suggestion, we rephrase as “Free energy profiles over STG8 (B) and LT ( **C**) connecting the solvent with the distal site through the cavities (trHb : CO) _1_, (trHb : CO) _2_ and (trHb : CO) _3_, for wild type (green), VG8F (orange) and VG8W (violet) mutant Mt-trHbN.”
- *References: page 9*
*Reference (21) is missing the title*
  We thank the reviewer; the reference was modified
